# *In Vitro* and *In Vivo* Antileishmanial Activity of Thioridazine

**DOI:** 10.1007/s11686-023-00746-2

**Published:** 2023-12-09

**Authors:** Sergio Sifontes-Rodríguez, Niurka Mollineda-Diogo, Lianet Monzote-Fidalgo, Alma Reyna Escalona-Montaño, José Antonio Escario García-Trevijano, María Magdalena Aguirre-García, Alfredo Meneses-Marcel

**Affiliations:** 1 División de Investigación, Facultad de Medicina, Unidad de Investigación UNAM-INC, Instituto Nacional de Cardiología Ignacio Chávez, Mexico City, Mexico; 2 Centro de Bioactivos Químicos, Universidad Central “Martha Abreu” de Las Villas, Santa Clara, Villa Clara Cuba; 3 Instituto de Medicina Tropical “Pedro Kourí” (IPK), Havana, Cuba; 4 Facultad de Farmacia, Universidad Complutense de Madrid, Madrid, Spain

**Keywords:** Thioridazine, *Leishmania amazonensis*, *Leishmania mexicana*, *Leishmania major*, Leishmaniasis

## Abstract

**Introduction:**

Leishmaniasis is a neglected disease with high prevalence and incidence in tropical and subtropical areas. Existing drugs are limited due to cost, toxicity, declining efficacy and unavailability in endemic places. Drug repurposing has established as an efficient way for the discovery of drugs for a variety of diseases.

**Purpose:**

The objective of the present work was testing the antileishmanial activity of thioridazine, an antipsychotic agent with demonstrated effect against other intracellular pathogens.

**Methods:**

The cytotoxicity for mouse peritoneal macrophages as well as the activity against *Leishmania amazonensis*, *Leishmania mexicana* and *Leishmania major* promastigotes and intracellular amastigotes, as well as in a mouse model of cutaneous leishmaniasis, were assessed.

**Results:**

Thioridazine inhibited the *in vitro* proliferation of promastigotes (50% inhibitory concentration—IC_50_—values in the range of 0.73 µM to 3.8 µM against *L. amazonensis*, *L. mexicana* and *L. major*) and intracellular amastigotes (IC_50_ values of 1.27 µM to 4.4 µM for the same species). In contrast, in mouse peritoneal macrophages, the 50% cytotoxic concentration was 24.0 ± 1.89 µM. Thioridazine inhibited the growth of cutaneous lesions and reduced the number of parasites in the infected tissue of mice. The dose of thioridazine that inhibited lesion development by 50% compared to controls was 23.3 ± 3.1 mg/kg and in terms of parasite load, it was 11.1 ± 0.97 mg/kg.

**Conclusions:**

Thioridazine was effective against the promastigote and intracellular amastigote stages of three *Leishmania* species and in a mouse model of cutaneous leishmaniasis, supporting the potential repurposing of this drug as an antileishmanial agent.

## Introduction

The term “leishmaniasis” encompasses a group of clinically distinct diseases caused by over 20 protozoan species of the genera *Leishmania and Endotrypanum* [1]. Leishmaniasis is important not only for the high incidence, prevalence and mortality, but also due to its disabling effect. An estimated 700,000 to 1 million new cases occur annually. However, beyond the suffering and lost human lives, the disease is an economical burden for the poorest sectors, which are the most vulnerable population [2, 3].

Pentavalent antimony compounds were the drugs of choice for several decades and are the first line drugs in many countries today. Amphotericin B, pentamidine, paromomycin (either injectable or topical) and miltefosine are the main therapeutic alternatives. In general, drug toxicity, cost, parasite resistance and the variable efficacy (depending on the nosogeographical entity) have limited the impact of treatment at the individual level and as a control measure. Therefore, the development of new, less toxic, more efficacious, and affordable antileishmanial drugs is considered a need [4–6].

The cost and time needed for the development of a new chemical entity to the final registration as a drug have been estimated to over US$ 2000 million and 12–15 years and the chances of returning such high investment in the context of a drug indicated for a disease that primarily affects poor countries are remote [7, 8].

Drug repositioning (finding new uses to already licensed drugs for other indications or drugs that have failed clinical trials due to efficacy issues) has emerged as a cost-effective alternative for the development of drugs for neglected diseases [9, 10]. In addition, a repositioned drug may advance quickly from *in vitro* and *in vivo* activity assays to its nomination as a drug candidate and to safety and efficacy clinical trials. Moreover, the cost of development has been estimated to be reduced in about US$ 320 million [11]. The likelihood of failure at the latest phases of development, which are the most expensive ones, also decreases since most of the pharmacology and safety profile of the compound is known in advance.

Current antileishmanial drugs including miltefosine (primarily developed as anticancer drug), amphotericin B (antifungal drug), pentamidine (antifungal—treatment of pneumonia caused by *Pneumocystis jiroveci*) and paromomycin (antibiotic), together with others that have demonstrated antileishmanial activity at experimental level [12–14], support the potential of drug repositioning for leishmaniasis.

Thioridazine is a first-generation phenothiazine antipsychotic drug that has gained attention in recent years as a repurposed drug for the treatment of cancer [15, 16], as well as bacterial [17, 18] and mycobacterial infections [19, 20].

Besides its intrinsic antimicrobial activity, thioridazine inhibits efflux pumps that mediate drug resistance in a number of microbes. For this reason, thioridazine has been tested in combination with other drugs in order to either potentiate drug effect or to revert drug resistance [16, 21]. As examples, thioridazine acts in synergy with the beta-lactam antibiotic, dicloxacillin, to kill methicillin-resistant *Staphylococcus aureus* *in vitro* and in a mouse model of peritonitis [22–24]. Molecular dynamics simulations suggest that such effect is probably mediated by inhibition of *S. aureus* efflux pump NorA [24]. Thioridazine increases the intracellular accumulation of the efflux pump substrate ethidium bromide in *Mycobacterium smegmatis* and *Mycobacterium avium* and inhibits the intrinsic efflux pump system of *M. avium* that causes erythromycin resistance [25]. In *M. tuberculosis*, thioridazine inhibits the respiration [26] and the efflux of rifampicin, a process principally mediated by proton gradient-dependent transporters [27].

Thioridazine is also active against *Trypanosoma cruzi* *in vitro* and in animal models [28]. In *Leishmania braziliensis*, *Leishmania guyanensis*, and *Leishmania mexicana*, thioridazine inhibits the energy-dependent efflux of pirarubicin and calcein cetoxymethylester, which are both well-known substrates of the mammalian multidrug-resistant pumps [29]. However, to the best of our knowledge, there is no previous report on the antileishmanial activity of thioridazine. Therefore, the aim of the present work was studying the *in vitro* inhibitory activity of thioridazine against the promastigote and intracellular amastigote stages of three *Leishmania* species as well as in mouse experimentally infected with *Leishmania amazonensis.*

## Materials and Methods

### Parasites and Cultures

*L. amazonensis* MHOM/BR/77/LTB0016 reference strain was kindly donated by the Department of Immunology of Fundação Oswaldo Cruz (Fiocruz), Brazil. *L. mexicana* MNYC/BZ/62/M379 and *L. major* MHOM/IL/81/Friedlin reference strains were kindly donated by Paul A. Bates, Division of Biomedical and Life Sciences, Faculty of Health and Medicine, Lancaster University, United Kingdom. The promastigotes were cultivated at 26 °C in Schneider’s Insect Medium (Sigma-Aldrich, St. Louis, MO, U.S.A.) supplemented with 10% heat-inactivated (56 °C, 30 min) fetal bovine serum (Gibco, USA) and antibiotics (200 UI penicillin and 200 µg/mL streptomycin). Exponential multiplication of the promastigotes of the three species was attained by passages every 3–4 days. Intracellular amastigotes were multiplied in mouse peritoneal macrophages obtained by peritoneal lavage of BALB/c mice and cultivated in RPMI-1640 medium supplemented with fetal bovine serum and antibiotics as described for Schneider’s Insect Medium.

### Test Compound

Thioridazine was supplied by BioCubaFarma, (Havana, Cuba) and was part of a commercial lot of pharmacologically active ingredient used to produce tablets. The sample satisfied quality specifications, was accompanied by the quality certificate and had 99.7% purity. For *in vitro* studies, the compound was dissolved in dimethyl sulfoxide (DMSO, Sigma-Aldrich, St. Louis, MO, USA) at 10 mg/mL and then serially diluted in an appropriate concentration range depending on its activity and the type of assay. For *in vivo* experiments, thioridazine was dissolved in 0.9% saline solution. Amphotericin B deoxycholate (Gibco, USA) was dissolved in sterile distilled water and used as positive control of activity for *in vitro* and *in vivo* assays.

### Activity Against Promastigotes

The growth inhibition assay was conducted according to Bodley and Shapiro’s procedure [30]. Briefly, all wells in the 96-well culture plate were seeded with 199 µL of culture medium containing 5 × 10^5^ stationary phase promastigotes/mL. One microliter of compound solutions in DMSO was then added per well, the plate was sealed with parafilm and incubated for 72 h at 26 °C. Control wells were treated with 1 µL DMSO. After the incubation period, 20 μL of 20 mg/mL p-nitro-phenyl-phosphate (Sigma-Aldrich) in sodium acetate (pH 5.5)—1% Triton X100 was added to each well. Plates were incubated for 3 h at 37 °C and absorbance was read in a Tecan Infinite 200 Pro microplate reader at 405 nm. Fifty percent inhibitory concentrations (IC_50_) were estimated by non-linear fitting to the sigmoid Emax equation [31]. Each drug concentration was tested in quadruplicate and the assay was repeated three times.

### Cytotoxicity Assay in Mouse Peritoneal Macrophages

BALB/c mice were killed by CO_2_ inhalation and their peritoneal macrophages were collected by washing the abdominal cavity with cold RPMI-1640 culture medium supplemented with antibiotics (sodium penicillin 200 UI and streptomycin 200 µg/mL) and 10% fetal bovine serum. The macrophages were distributed in 96-well culture plates at 10^5^ cells/well. After 4 h incubation at 37 °C and 5% CO_2_, the culture medium was replaced by fresh medium containing test compounds and the plates were incubated for other 48 h. Afterwards, 10 µL Alamar Blue (DAL1025, Thermo Fisher Scientific) was added per well. After other 6–8 h of incubation (37 °C and 5% CO_2_), the reduction of Alamar Blue by viable cells was assessed by reading fluorescence at EX/EM 530/585 nm (cutoff, 550 nm) in a Tecan Infinite 200 Pro microplate reader. Fifty percent cytotoxic concentrations (CC_50_) were estimated by non-linear fitting to the Emax sigmoid model [31].

### Drug Activity Against Intracellular Amastigotes

Mouse peritoneal macrophages were collected, seeded in 96-well culture plates at 10^5^ cells/well and incubated at 34 °C, 5% CO_2_ for 2 h. Non-adherent cells were removed by change of the culture medium and the adhered cells were infected with stationary phase promastigotes (either *L. amazonensis*, *L. mexicana* or *L. major* promastigotes) at a rate of four parasites per host cell for *L. amazonensis* and ten parasites per macrophage for the other two species. After 4 h of incubation at 34 °C and 5% CO_2_, free promastigotes were eliminated, and the medium was replaced by 199 µL culture medium and 1 µL test compound solutions in DMSO. Four replicates of each drug concentration were tested in every assay. Plates were then incubated for 72 h at 37 °C and 5% CO_2_. Afterwards, the culture medium was discarded, replaced by Schneider’s Insect Medium and the plates were incubated for other 72 h at 26 °C to allow surviving amastigotes to transform into promastigotes and replicate. P-nitro-phenyl-phosphate was then added to each well as described in the promastigote assay and the IC_50_ values were estimated as previously indicated.

### Animals

Female, 16–18 g, 6–8-weeks-old BALB/c mice were supplied by the National Center for the Production of Laboratory Animals (Cuba). They were maintained under controlled environmental conditions (room temperature 22–25 °C, relative humidity 60–65%, light cycle 10 h light-14 h dark) and were handled by qualified personnel. At the end of studies, mice were killed by CO_2_ inhalation. The experimental protocol was approved by the Institutional Ethics Committee for the Care and Use of Laboratory Animals of the Center of Bioactive Chemicals (CBQ/CEAE/2021.4).

### *In Vivo* Antileishmanial Assay

Fifty mice were infected by intradermal inoculation in the left hind footpads with 10^7^ stationary phase *L. amazonensis* promastigotes. Once lesions developed (21 days post-infection), mice were randomly allocated to five experimental groups and were treated for 14 days. Two groups were treated with thioridazine (50 or 25 mg/kg, intraperitoneal route); one with amphotericin B (5 mg/kg, intraperitoneal route, every other day); one with 0.9% saline solution (0.2 mL, intraperitoneal route); and one group received no-treatment at all (control group).

The dorsoplantar diameters of both rear limbs were weekly measured using a caliper (Krœplin, Längenmesstechnick, error 0.05 mm). Lesion size was calculated by subtracting the measure of the non-infected pad to that of the infected one. The groups were statistically compared by repeated measures analysis of variance, the Fisher’s least significant difference test and the Dunnett’s test, using STATISTICA software [32].

Four mice per group were killed 2 weeks after the end of therapy and the number of amastigotes in the infected tissue was determined by the limiting dilution assay technique as described elsewhere [33]. Parasite loads were compared by Kruskal–Wallis test and the distribution-free multiple comparisons test. Values of *p* under 0.05 were considered statistically significant.

### Dose–Effect of Thioridazine Against Experimental Cutaneous Leishmaniasis

Experimentally infected mice (as described above) with evident lesions were randomized and then treated by intraperitoneal route for 14 days with either thioridazine at 25, 20, 15, 10 or 5 mg/kg; amphotericin B at 5 mg/kg or were not treated at all (control). The lesion size and parasite loads were assessed and statistically analyzed as described for the previous assay. The doses reducing lesion size and parasite load by 50% compared to controls were estimated by non-linear fitting of dose–effect curves.

## Results

### *In Vitro* Antileishmanial Activity and Cytotoxicity

Thioridazine was active against *L. amazonensis*, *L. mexicana and L. major* promastigotes (Table 1). IC_50_ values ranged from 1.73 µM to 3.8 µM and *L. amazonensis* seamed slightly more sensitive than the two other species. It was also active against intracellular amastigotes of the three species of *Leishmania* tested. The IC_50_ values against intracellular amastigotes ranged from 1.27 to 4.4 µM.

**Table 1:** *In vitro* growth inhibitory activity and cytotoxicity of thioridazine against three *Leishmania* species

Stage	*Leishmania* species	IC_50_ (µM)	
		Thioridazine	Amphotericin B
Promastigotes	*L. amazonensis*	1.73 ± 0.13	0.029 ± 0.005
	*L. mexicana*	3.06 ± 1.13	0.046 ± 0.012
	*L. major*	3.80 ± 1.38	0.020 ± 0.007
Amastigotes	*L. amazonensis*	1.27 ± 0.27	0.033 ± 0.014
	*L. mexicana*	3.1 ± 0.52	0.036 ± 0.008
	*L. major*	4.4 ± 0.41	0.031 ± 0.003
	Average	2.92 ± 1.57	0.033 ± 0.003
Mouse peritoneal macrophages (CC_50_-µM)		24.0 ± 1.89	1.38 ± 0.26
Selectivity index*		8.2	41.8

*SI* Selectivity index. The selectivity index was calculated by dividing the 50% cytotoxic concentration in mouse peritoneal macrophages by the average IC_50_ in either promastigotes or intracellular amastigotes. All values are the mean ± standard deviation of three replicated assays

Thioridazine was moderately cytotoxic for the host mammalian cells (mouse peritoneal macrophages); however, CC_50_ values were significantly higher than the IC_50_ values against the intracellular amastigotes for the three *Leishmania* species tested.

### Efficacy in a Murine Model of Cutaneous Leishmaniasis

Dose-finding pilot studies (data not shown) were conducted prior to the activity assays to identify maximum tolerable doses (MTD). Thioridazine was daily administered by intraperitoneal route for 21 days and the MTD was 50 mg/kg. Higher doses of thioridazine (100 mg/kg) caused a severe and lethal lethargy of mice.

**Fig. 1:**
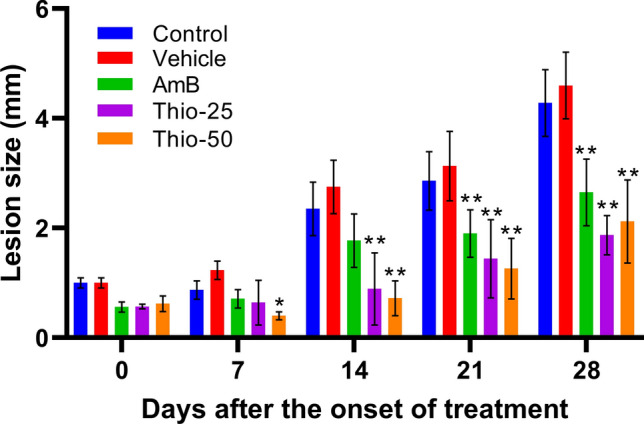
Reduction of lesion growth in mice experimentally infected with *L. amazonensis* and treated with thioridazine. *p < 0.05, **p < 0.01 (compared to the control group)

In the first *in vivo* activity study, thioridazine was administered at its MTD and at half the MTD, i.e., 50 mg/kg and 25 mg/kg (Fig. 1). At both dose levels, it was observed a statistically significant arrest of lesion growth compared to non-treated mice (*p* < 0.01). It is relevant that the effect of thioridazine was evident since the first week of treatment (50 mg/kg, *p* < 0.05), whereas amphotericin B required 3 weeks of treatment to demonstrate a visible and statistically significant effect.

**Fig. 2:**
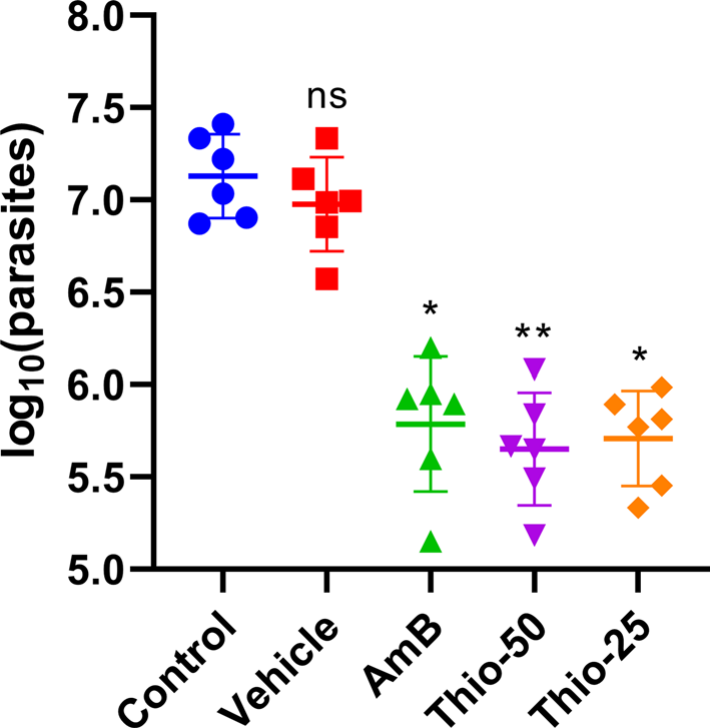
Effect of thioridazine treatment on the number of parasites at the lesion site of mice experimentally infected with *L. amazonensis.* *p < 0.05, **p < 0.01, ns: *p* > 0.05 (compared to the control group). AmB: amphotericin B, Thio: thioridazine

The number of parasites in the infected skin tissue 2 weeks after the end of therapy (Fig. 2) was in correspondence with lesion size. Mice treated with thioridazine at 50 mg/kg and 25 mg/kg showed 96.7% and 96.2% reduction of parasite load, respectively, compared to controls (*p* < 0.01) and were similar (*p* > 0.1) to amphotericin B-treated mice (95.5% reduction). The effect of thioridazine at the two dose levels was similar (*p* > 0.1) both in terms of lesion size and parasite load.

**Fig. 3:**
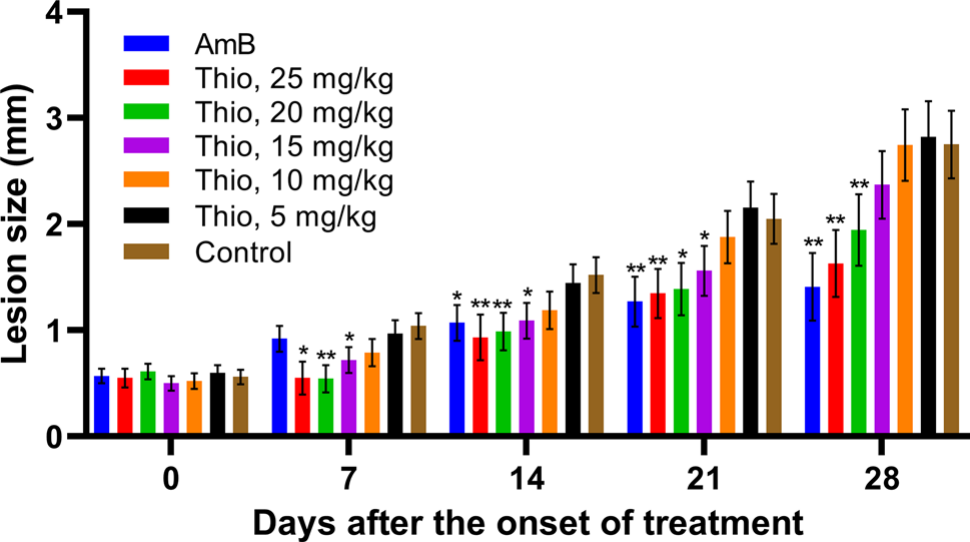
Dose–effect of thioridazine on the lesion size of mice experimentally infected with *L. amazonensis*. *p < 0.05, **p < 0.01. The estimated ED_50_ (mean ± standard error) at 21 days was 23.3 ± 3.1 mg/kg and 24.1 ± 2.9 mg/kg at 28 days

**Fig. 4:**
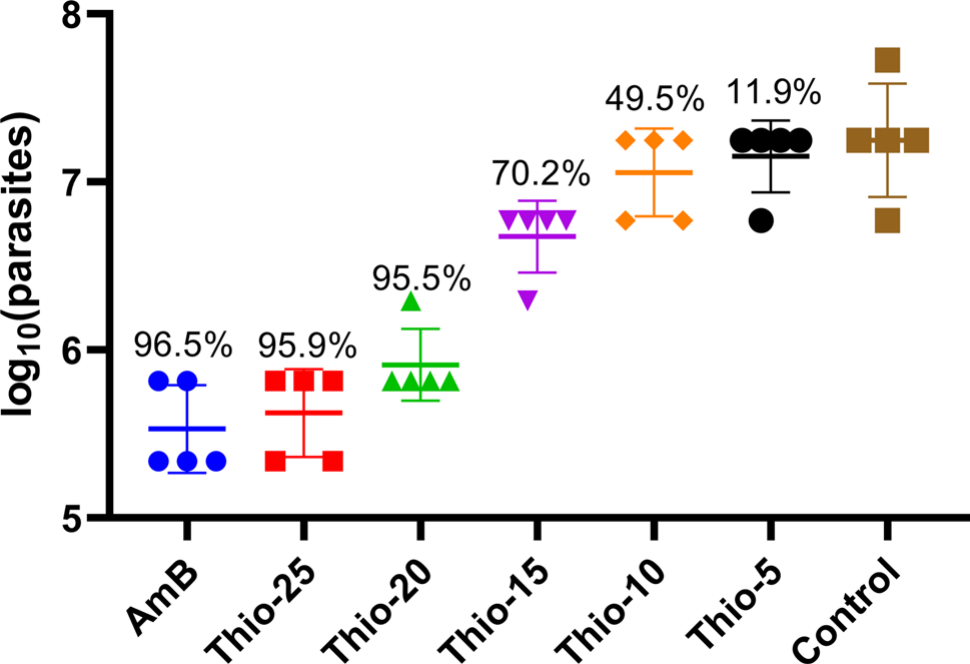
Effect of thioridazine dose on the amount of parasite in lesions. Data labels indicate percent reduction in parasite load relative to untreated control mice. Bars depict geometric means and error lines indicate min and max values

In the second animal efficacy study, thioridazine was administered at doses from 5 to 25 mg/kg (Fig. 3). A statistically significant effect on lesion size compared to control mice was achieved with doses from 15 to 25 mg/kg. The estimated 50% effective dose (ED_50_; mean ± standard error) at 21 days after the onset of treatment was 23.3 ± 3.1 mg/kg and 24.1 ± 2.9 mg/kg at 28 days. Comparable results were obtained in terms of parasite loads at 28 days (Fig. 4), however, with a lower ED_50_ of 11.1 ± 0.97 mg/kg.

## Discussion

Thioridazine is a piperazine phenothiazine derivative which acts by postsynaptic inhibition of dopamine receptors. Thioridazine has other peripheral and central nervous system effects, producing both alpha-adrenergic stimulation and blocking histamine- and serotonin-mediated effects. Thioridazine is indicated for the therapy of acute and chronic psychosis. It was approved for use in the United States in 1978 and was formerly a commonly prescribed antipsychotic medication, but in recent years has been replaced in large part by the atypical antipsychotics, which have fewer extrapyramidal side effects. Use of thioridazine is also restricted because of its propensity to cause prolongation of the QTc interval and increased risk of sudden death. The recommended dose in adults is 50 mg to 100 mg three times daily, increasing based upon effect and tolerance to a maximum of 800 mg daily [34]. Despite its side effects, thioridazine has been the recent focus of repurposing as an anticancer drug [15, 16, 35, 36], as an antibacterial [17–20, 37, 38] and as an antiviral [39].

To our knowledge, there is no previous report on the antileishmanial activity of thioridazine neither *In vitro* (promastigotes and amastigotes) nor *in vivo*. However, it is known that some phenothiazine derivatives, thioridazine inclusive, can inhibit energy-dependent efflux systems in *L. guyanensis*, *L. braziliensis* and *L. mexicana* promastigotes [29]*.*

In the present study, low concentrations of thioridazine inhibited proliferation of *In vitro* cultured promastigotes and amastigotes. The activity was specific, as indicated by appropriate therapeutic indices in both the extracellular and the intracellular *In vitro* systems. Thioridazine was also capable of reducing the growth of lesions and the load of parasites in the skin of mice experimentally infected with *L. amazonensis*. Furthermore, the *in vivo* effect of thioridazine was comparable to that of amphotericin B at 5 mg/kg used as positive control.

Thioridazine showed a dose-dependent and statistically significant effect *in vivo* at doses from 15 to 25 mg/kg. The maximum recommended dose in humans is 800 mg/day (about 11.4 mg/kg for a patient of 70 kg) [34, 40], which is allometrically equivalent to 137 mg/kg in mice [41]. According to these figures, thioridazine could be a relatively safe systemic alternative treatment for cutaneous leishmaniasis. However, daily oral doses above 50 mg/kg caused a severe respiratory depression in mice and 100 mg/kg were lethal.

Though thioridazine was widely used for years as an antipsychotic agent (and it is still used in many countries), its indication as an oral drug for cutaneous leishmaniasis could raise ethical concern, due to the potential occurrence of severe side effects in the context of a nonlethal form of the disease. Consequently, developing a formulation of thioridazine for topical used could be more reasonable. Given its molecular weight (370.6 g/mol) and lipophilicity (logK_o/w_ = 5.90) values, the development of a formulation with proper skin permeability is theoretically feasible according to the values of permeability constant (1.2–4.3 × 10^–5^ cm/s) calculated using three different QSAR models [42–44].

Like *Leishmania* amastigotes, *M. tuberculosis* resides inside macrophages. Accessing inner cell compartments could be a problem for other drugs, but thioridazine. This drug accumulates inside macrophages and reaches higher intra-macrophagic concentration compared to that in blood plasma [45, 46]. The *In vitro* activity of thioridazine against *M. tuberculosis*, its efficacy as monotherapy and in combination with anti-tuberculosis drugs in animal models, as well as in extensively drug-resistant tuberculosis patients have been reported [47, 48]. Thioridazine acts synergistically with anti-tuberculosis drugs *in vivo*, particularly against drug-resistant *M. tuberculosis* strains [45, 49]. Based on such results, the second use of thioridazine as a drug for the treatment of tuberculosis has been proposed.

Thioridazine improves cardiac function and survival time in acute and chronic murine models of Chagas’ disease [28]. Moreover, phenothiazine derivatives also inhibit two major *T. cruzi* enzymes: trypanothione reductase and dihydrolipoamide dehydrogenase [50, 51]. Due to the close taxonomic relationship between *Leishmania* spp. and *T. cruzi*, thioridazine probably inhibits the homologous enzymes in *Leishmania* spp.

Efflux pumps not only participate in drug extraction from the cell and mediate drug resistance [17, 27, 29] but they are also involved in the physiological detoxication of the cell by preventing the accumulation of harmful metabolic byproducts [16, 52]. This way, efflux pump inhibition by thioridazine potentially mediates its direct antileishmanial effect by promoting the intracellular accumulation of waste products.

In conclusion, the results demonstrated the potential of thioridazine as an antileishmanial drug, which is further supported by thioridazine activity against intracellular pathogens such as *T. cruzi* and *M. tuberculosis*. Aspects like its activity in murine models of visceral leishmaniasis, the interaction with currently used antileishmanial drugs (specially in drug-resistant strains) and the antileishmanial activity of potential neurologically inert thioridazine metabolites (thioridazine-5-sulphoxide) [46] and derivatives as well as nanoencapsulated thioridazine [53] deserve additional investigation.

## Data Availability

Primary data supporting the results are available upon request to the corresponding author.
